# Cerebral Perfusion and Neuromonitoring during Complex Aortic Arch Surgery: A Narrative Review

**DOI:** 10.3390/jcm12103470

**Published:** 2023-05-15

**Authors:** Andrea Montisci, Giulia Maj, Corrado Cavozza, Andrea Audo, Stefano Benussi, Fabrizio Rosati, Sergio Cattaneo, Lorenzo Di Bacco, Federico Pappalardo

**Affiliations:** 1 Division of Cardiothoracic Intensive Care, Cardiothoracic Department, ASST Spedali Civili, 25123 Brescia, Italy; 2 Cardiothoracic and Vascular Anesthesia and Intensive Care Unit, AO SS. Antonio e Biagio e Cesare Arrigo, 15121 Alessandria, Italy; 3 Department of Cardiac Surgery, AO SS. Antonio e Biagio e Cesare Arrigo, 15121 Alessandria, Italy; 4 Division of Cardiac Surgery, Cardiothoracic Department, ASST Spedali Civili and University of Brescia, 25123 Brescia, Italy

**Keywords:** aortic surgery, innominate artery, hypothermic circulatory arrest, near-infrared spectroscopy, cerebral perfusion, cerebral protection

## Abstract

Complex ascending and aortic arch surgery requires the implementation of different cerebral protection strategies to avoid or limit the probability of intraoperative brain damage during circulatory arrest. The etiology of the damage is multifactorial, involving cerebral embolism, hypoperfusion, hypoxia and inflammatory response. These protective strategies include the use of deep or moderate hypothermia to reduce the cerebral oxygen consumption, allowing the toleration of a variable period of absence of cerebral blood flow, and the use of different cerebral perfusion techniques, both anterograde and retrograde, on top of hypothermia, to avoid any period of intraoperative brain ischemia. In this narrative review, the pathophysiology of cerebral damage during aortic surgery is described. The different options for brain protection, including hypothermia, anterograde or retrograde cerebral perfusion, are also analyzed, with a critical review of the advantages and limitations under a technical point of view. Finally, the current systems of intraoperative brain monitoring are also discussed.

## 1. Introduction

Complex ascending and aortic arch surgery with open distal anastomosis requires circulatory arrest (CA) to obtain a bloodless operating field. This strategy entails the interruption of the normal arterial blood supply to the brain and requires the implementation of cerebral perfusion strategies [1].

Neurological complications are associated with increased mortality, longer hospitalization, healthcare resources’ utilization and impaired quality of life [2,3].

The technical improvement of extracorporeal circulation, increased knowledge of the pathophysiology of cardiopulmonary bypass and special organ protection strategies have helped to reduce the incidence of complications and death to an acceptable rate in aortic surgery [3].

The etiology of these complications is most likely multifactorial, involving cerebral embolism, hypoperfusion, hypoxia and inflammatory response, eventually leading to a regional or global imbalance between cerebral oxygen demand and supply [4].

In this article, we will provide an overview of all the aspects regarding complex ascending and aortic arch surgery, including the pathophysiology of cerebral ischemia, cannulation techniques for the conduction of cardiopulmonary bypass (CPB), brain perfusion strategies and intraoperative monitoring of brain function.

## 2. Brain Damage after Ascending Aorta and Aortic Arch Surgery: General Considerations

The efficacy of cerebral protection during ascending aorta and aortic arch surgery is still the most important determinant of favorable postoperative neurologic outcomes both in elective and urgent/emergent aortic arch surgery [5]. Neurologic events range from 3.4% in elective aortic arch operations to 12% in acute Stanford type A dissections [6,7]. Although recent studies have consistently demonstrated a relatively low incidence of stroke (between 2% and 8%) following aortic surgery with hypothermic CA, new postoperative cerebral lesions are frequently observed when neuroimaging is obtained [8,9].

These silent lesions may play a role in the development of cognitive and neuropsychological disorders, which have a significant impact on the postoperative course and patients’ morbidity [10].

Among the different cerebral protection strategies, deep hypothermia with complete CA has been considered the gold standard for years, but there is always concern about the “safe” duration of cold ischemia of the brain, the deleterious systemic effects of profound hypothermia and the sequelae of the prolonged CPB time required to cool and then rewarm the patient [11,12].

## 3. Pathophysiology

The human brain weighs approximately 1400 grams (g) and represents only 5% of one’s body weight but receives approximately 15% of the total cardiac output [3].

The cerebral metabolic rate of oxygen (CMRO_2_), according to Mc Coullugh, can be calculated with this formula:

CMRO_2_ = CBF × cerebral AV oxygen content difference/100

where CBF is cerebral blood flow and artero-venous difference is the difference in O_2_ content between the arterial and venous cerebral blood.

The cerebral blood flow (CBF) is autoregulated for a wide range of blood pressure values, meaning that from 50 to 150 mmHg of systolic blood pressure, the cerebral blood flow remains quite constant, due to local mechanisms of vascular tone adaptation [13].

The loss of autoregulation assumes that cerebral blood flow becomes dependent on blood pressure, and it is observed that when systolic blood pressure falls below 50 mmHg, blood temperature falls below 12 °C and blood flow is between 20 to 50 mL/100 g/min of the cerebral tissue. Overt ischemia arises when cerebral blood flow falls below 20 mL/100 g/min of the cerebral tissue; when CBF falls below the critical value of 10 mL/100 g/min, neuronal death happens [14].

Ischemia tolerance is highly increased by hypothermia. Indeed, at a normal body temperature (36.1–37.0 °C), the oxygen consumption of the human brain is 2.9 mL/g/min; however, at 25 °C, the oxygen consumption is reduced to 0.9 mL/g/min, and at 20 °C it is 0.2 mL/g/min [3].

## 4. Aetiology of Neurologic Injury during Aortic Surgery

Neurological damage after cardiac surgery can be classified according to Roach’s

classification, that distinguishes two types of injury:

1. Type 1: including death from stroke and non-fatal stroke, transient ischemic attack (TIA), hypoxic encephalopathy, stupor and coma;
2. Type 2: includes cognitive decline, delirium, memory loss and epilepsy without focal deficit [3].

Ergin’s classification introduced the difference between a neurological injury characterized by a transient clinical expression versus a permanent one [3,15].

After cardiac surgery, the main pathogenetic mechanisms of stroke are represented by emboli, accounting for 60% of cases, followed by hypoperfusion (10%) [16]. Therefore, multiple players contribute to the final outcome: extracorporeal perfusion-related events, temperature management, blood flow, cerebral perfusion techniques, (anterograde versus retrograde, unilateral versus bilateral), pharmacologic protection, aortic cross-clamping, temperature and duration of the deep hypothermic circulatory arrest (DHCA) [17,18,19].

Two main aspects are relevant for cerebral protection during aortic surgery:

(a) The strategy for cerebral perfusion on top of hypothermic circulatory arrest.
(b) Cerebral monitoring techniques associated with possible therapeutic interventions.

## 5. Strategy for Cerebral Perfusion on Top of Hypothermic Circulatory Arrest

The current practices for proximal aortic arch surgery with regards to neuroprotection strategies include:

i. Deep hypothermic circulatory arrest (DHCA);
ii. Retrograde cerebral perfusion (RCP) with DHCA;
iii. Antegrade cerebral perfusion (ACP) with DHCA;
iv. ACP with moderate hypothermic circulatory arrest.

All these strategies are currently employed. A recent review of the STS database, on 2982 cases of emergent aortic surgery [20], showed that hypothermic circulatory arrest (HCA) was used in 78% of patients. Among those undergoing HCA, brain strategies included ACP (31%), RCP (25%), both (4%) and none (40%).

Current trends suggest that ACP techniques are increasingly being used for brain protection with less reliance on hypothermia alone. Indeed, ACP improves the neurological outcome even more in the case of prolonged HCA [21]. In this regard, various sites of cannulation permit the institution of ACP: the axillary artery, the innominate artery and the left common carotid artery cannulation.

### 5.1. Axillary Artery Cannulation (Table 1)

Axillary artery cannulation has demonstrated several advantages over aortic and femoral cannulation in aortic surgery [1,22,23]. This strategy allows the preservation of the antegrade flow in the aortic arch and in the descending aorta. The physiological blood flow pattern directed to the brain is associated with improved neurological protection both in acute type A aortic dissection and in elective aortic arch surgery, lowering the risk for embolization into right-sided cerebral vessels.

**Table 1:** Summary of different cannulation strategies’ pros and cons during aortic surgery.

Cannulation Site	PROs	CONs
Femoral Artery	Cannulation first (before sternotomy) Easy and fast approach Two sites of cannulation Avoids dissected aorta manipulation	Retrograde perfusion Risk of dissecting vessels Risk of contamination
Right Axillary Artery	Cannulation first (before sternotomy) Anterograde aortic perfusion Anterograde cerebral perfusion No cannula switch Seldom dissected Direct cannulation or side branch technique	Time consuming Fragile vessel Difficult isolation in obese patients Vessel and nerve injury
Left Axillary Artery	Cannulation first (before sternotomy) Anterograde aortic perfusion Posterior cerebral circle anterograde perfusion (left vertebral artery) No cannula switch Seldom dissected Direct cannulation or side branch technique	Time consuming Fragile vessel Difficult isolation in obese patients Vessel and nerve injury No complete cerebral perfusion
Carotid Arteries	Two sites of cannulation Fast and simple isolation Cannulation first (before sternotomy) Anterograde aortic perfusion Cerebral perfusion No cannula switch Seldom dissected	In case of small lumen, do not support high CPB flows
Innominate/Subclavian Artery	Easy and direct Anterograde cerebral perfusion No cannula switch Antegrade aortic perfusion	Dissected vessel Sternotomy first
Ascending Aorta “true lumen”	Antegrade flow	Sternotomy first Not suitable for direct cerebral perfusion (cannula switch) Risk of false lumen cannulation Technically demanding in case of totally dissected aorta

Secondly, the axillary cannula avoids the “sandblast effect” of turbulent flow from the cannula tip in aortic cannulation. The turbulent high-speed flow of the aortic cannula may have a negative impact on atheroma and calcifications, causing plaques’ disaggregation and cerebral embolism.

Thirdly, when circulatory arrest is required, selective antegrade cerebral perfusion (sACP) is obtained with a single cannula, instead of two.

Furthermore, sACP is never discontinued, and continuous back flow in all aortic arch vessels minimizes the risk of air embolization to the brain [17].

Finally, the axillary cannulation does not interfere with the surgeon’s open distal anastomosis, while the lower body is kept hypothermic during CA.

Although this perfusion strategy is the choice of many centers worldwide, axillary artery cannulation has been associated with some local complications such as brachial plexus injuries, arm ischemia, arm hyperperfusion and seroma formation [24].

The artery may be approached immediately under the right clavicle, the first portion of the axillary artery or in its second portion delto-pectoral sulcus. At this level, the vessel is rarely affected by atheromatous lesions or dissection. Accessing the axillary artery requires a separated incision, subcutaneous tissue dissection, partial division of the pectoralis major and minor muscles and dissection of the retromuscular connective tissue rounding the brachial plexus. The axillary artery may have different anatomical relationships with the axillary vein and its small tributaries that should be carefully ligated or cauterized in order to avoid further bleeding.

When the vein is anterior to the artery, the vein must be mobilized and surrounded, then gently pulled down. The artery is then exposed for 4–5 cm of its length.

The manipulation of the axillary artery has to be extremely careful due to its peculiar fragility. The local complication rate, reported with an incidence of 3.8% by a recent randomized controlled trial [25], can be reduced by the use of a side graft for axillary/subclavian cannulation, as shown by Sabik and colleagues [26].

Finally, as the axillary artery cannulation requires an additional incision, the total surgical time may be prolonged, making the procedure challenging and time-consuming in case of unstable hemodynamic conditions.

#### 5.1.1. Direct Cannulation Technique

The artery is controlled upstream of and downstream from the cannulation site by snares, and cross-clamped distally after heparinization. A 5/0 polypropylene purse-string suture is carried out on the anterior wall of the vessel and put on a tourniquet. A transverse or longitudinal (according to the surgeon’s habits and preference) small incision (about 5 mm) is made, through which a 14–18 French arterial cannula can be carefully placed into the arterial lumen, at a distance of 3–4 cm. Alternatively, the Seldinger technique can be employed: the artery is punctured with a hollow needle and a guidewire is advanced. Serial, progressive dilators open the way for the cannula that is eventually fixed to the vessel [2,23,27].

#### 5.1.2. Side-Arm Graft Technique

Vessel loops are placed to gain control proximally and distally, and the full dose of heparin required for the cardio-pulmonary bypass (CPB) is administered. An 8–10 mm Dacron graft is anastomosed in an end-to-side fashion to the axillary artery with a running 5/0 prolene suture. A 24 French straight cannula is inserted into the Dacron graft for arterial inflow [26,28]

#### 5.1.3. Right Subclavian Artery Cannulation without Infraclavicular Incision (Table 1)

**Figure 1:**
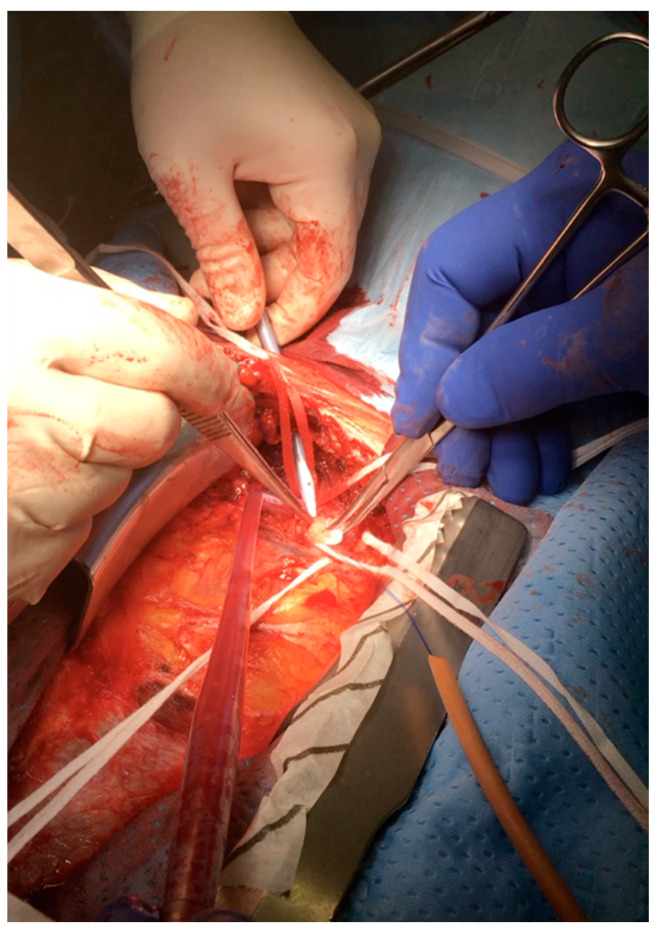
Right subclavian artery cannulation without infraclavicular incision with Seldinger technique.

A skin incision is performed and extended about 2 cm to the right neck (Figure 1). The thymus is completely dissected off the pericardium, and all the thymic veins, tributaries of the innominate vein, are divided, thus exposing the entire length of the extrapericardial ascending aorta up to the arch vessels. Before the pericardial incision is made, the arteries are identified. The innominate vein is isolated and retracted inferiorly with an umbilical tape. The innominate and right subclavian arteries are exposed and encircled with an umbilical tape (Figure 1). The proximal portion of the right subclavian artery arises from the bifurcation of the right brachiocephalic trunk, posteriorly to the right sternocostal joint, and is crossed by the vagus nerve, giving origin to the right recurrent laryngeal branch, which hooks around the right subclavian artery. Appropriate surgical exposure is essential in order to avoid the risk of vagus nerve injury [29]. The right subclavian artery, if not involved in the dissection, is gently retracted and a single purse-string suture is placed at least 1 cm away from its origin from the innominate artery. An arterial cannula is inserted using the Seldinger technique. Extreme carefulness should be adopted during purse-strings, and cannulation maneuvers should be adopted given the extreme fragility of this vessel, more than the femoral artery, to avoid iatrogenic traumas and dissections.

### 5.2. Innominate Artery Cannulation

Direct innominate artery (IA) cannulation has emerged as a simplified technique for ACP (Table 1).

**Figure 2:**
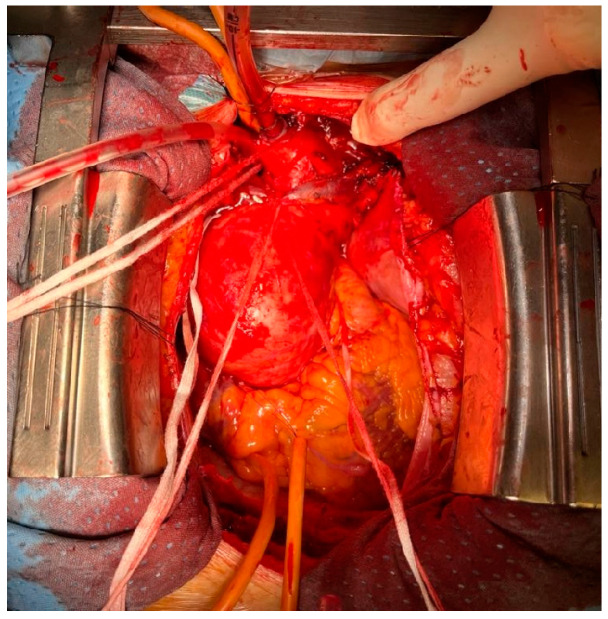
Innominate artery direct cannulation for systemic and cerebral perfusion.

The axillary artery might be time consuming and not straightforward in emergent situations. The IA can be accessed via a sternotomic incision extended a little cephalad and its surgical dissection is easier as compared to axillary artery dissection (Figure 2). Most patients are anatomically suitable for IA, with a low rate of complications such as arterial damage or dissection. Peterson et al. reported the first randomized trial comparing axillary to IA cannulation in the context of the elective repair of the ascending aorta and proximal arch surgery: they concluded the IA cannulation is non-inferior to axillary with regard to neurological outcomes [30]. However, the trial was focused specifically on elective proximal hemiarch surgery, leaving unanswered several questions regarding the outcomes in emergent aneurysm surgery, aortic dissection and more complex arch surgery. In their review of 2290 patients undergoing IA cannulation, they reported a 30-day mortality of 2.7% and an incidence of postoperative stroke of 1.25% [31]. Furthermore, IA could offer the advantage of reduced procedural and CPB times in obese patients. Chumwa et al. demonstrated a significantly lower total surgical time with IA cannulation (318 vs. 454 min, *p* < 0.001), though interinstitutional differences could have contributed to these findings [32].

Compared with the axillary artery cannulation, the IA has a lower flow resistance, and the cannulation site is always under control, with a reduced risk of blood loss in the operative field and/or kinking of the cannula. Indeed, Elderiry et al. demonstrated a significantly lower requirement for red blood cell (2 vs. 3 units, *p* < 0.001), platelet (1 vs. 2 units, *p* < 0.001) and fresh frozen plasma transfusion (2 vs. 6 units, *p* < 0.001) in IA versus axillary cannulation technique in an elective setting [33]. Contraindications to IA cannulation are very rare. A preoperative CT scan and/or epiaortic ultrasound allow the detection of atheromatous plaques and dissection at the site of cannulation. Hostile chest and obesity generally do not represent a contraindication to this approach. The length of the IA is also a key factor to ensure a safe cannulation 4–5 cm distal from the origin of the vessel, where the likelihood of atheromatous material dislodging and embolization is supposed to be lower [34]. The main limitations to its use for ACP during type A aortic dissection are the frequent involvement from the dissection.

In the case of dissection or shortness of the IA and a need for prompt CPB institution, Cavozza et al. suggested to perform the right subclavian artery cannulation without infraclavicular incision [35].

#### Surgical Technique

Following median sternotomy and full systemic heparinization, the innominate vein is mobilized with a vessel loop and retracted cephalad to allow the exposure of the IA. The base of the IA is then dissected and snared with an umbilical tape; a single purse-string suture is then placed on the anterior surface of the IA approximately 1 cm distal to its origin using a 5-0 Prolene suture. The arterial cannula is placed at a depth of only 1–1.5 cm into the vessel.

### 5.3. Left Common Carotid Artery

The surgical approach to the left carotid artery is feasible and very fast. It represents a quick option for an emergency, especially in obese patients. In experienced hands, 15 min are usually needed from the initial skin incision to the completion of arterial cannulation. The common carotid artery is larger than the axillary artery, with a stronger wall. The high compliance of the arterial vessel allows high CPB flow as in obese patients [17,36].

### 5.4. Ascending Aorta

Poor outcomes have been reported for the direct cannulation of the ascending aorta [11], due to the potential aortic dissection, rupture and false lumen perfusion. Therefore, it is usually avoided [11]. Recently, some advantages have been reported, especially in the case of hemodynamic instability. This technique could be performed safely under an epiaortic ultrasound-guided Seldinger technique, and it allows a quick establishment of anterograde perfusion and core cooling [37].

### 5.5. Femoral Artery Cannulation

Femoral artery cannulation has long been the standard site for peripheral cannulation in aortic surgery in non-elective cases. Retrograde aortic perfusion presents as a primary advantage over ACP in that it allows for a completely unencumbered arch reconstruction, with no obstructive clamps or cannulae within the field. The avoidance of direct manipulations of the ascending aorta and the good access to the vessel are advantageous for the surgical technique, but retrograde flow through a diseased abdominal and descending thoracic aorta increases the risk of cerebral emboli and malperfusion.

Moreover, retrograde perfusion against an aortic cross-clamp may cause perfusion of the re-entry tears, new re-entry tears and/or visceral and arch vessel malperfusion and an increased risk of neurological injury. For this reason, femoral cannulation has been gradually decreasing over the last 10 years [22].

## 6. Anterograde versus Retrograde Cerebral Perfusion

The optimal brain protection strategy during complex aortic surgery still remains a matter of debate. The use of DHCA alone is reported to be inferior when compared to cerebral perfusion strategies combined with DHCA [18,19]. Nowadays, anterograde cerebral perfusion (ACP) and retrograde cerebral perfusion (RCP) are well-established techniques to protect the brain during DHCA. However, both approaches have pros and cons. With the choice of ACP, low-flow volume cold blood is delivered to the brain through the arterial cerebral branches of the aortic arch, in order to maintain a nearly physiologic cerebral perfusion. Moreover, ACP provides an independent control of the temperature and/or flow to the cerebral and systemic circulation. The cannulation site, flow, pressure and temperature of the perfusate are highly varied among different studies (Table 2 and Table 3) [3].

With regard to the cannulation site, since the first description of bilateral normothermic carotid cannulation by Kazui et al. 38], the technique evolved significantly and currently includes different approaches, ranging from unilateral cerebral perfusion through the cannulation of the right axillary artery; bilateral cerebral perfusion through the cannulation of the right axillary artery and left carotid artery; bilateral cerebral perfusion through the direct cannulation of the left and right carotid arteries (Kazui technique) [17] (Table 4 and Table 5).

As regards the perfusion flow, there are different protocols that range from 8 to 16 mL/Kg/min, with most centers using 8–12 mL/kg/min and with a trend toward higher flows in warmer temperature protocols. Johnson et al. identified the threshold of 6 mL/Kg/min in a porcine model of ACP as the lowest value of perfusion not associated with biochemical evidence of brain damage [52]. At the same time, the use of a high flow rate can be associated with an increase in intracranial pressure, as shown by Haldenwang in a porcine model [53].

ACP should be preferred when a long time of DHCA is expected. However, ACP has the risk of embolic events and the risk of vascular injuries of cannulated vessels.

On the contrary, RCP administered from the superior vena cava avoids arch branches’ manipulation, reducing the risk of iatrogenic injuries due to selective cannulation and potentially flushing away air and embolic debris from the arterial side. Cerebral protection with RCP is reported to be safely administered for up to 80 min, but longer periods of RCP are reported to be negative prognostic factors for morbidity and mortality [54].

Some studies demonstrated that cerebral perfusion is maximized during RCP, keeping perfusion pressure between 20 and 25 mmHg [3,55]. However, when RCP is employed, there is no possibility of direct monitoring of the cerebral perfusion pressure. Nevertheless, RCP may potentially cause cerebral edema if the perfusion pressure exceeds 30 mmHg [56], exacerbating cerebral injury. In addition, there is a risk of a veno-venous shunt from the cerebral to systemic venous system, potentially reducing brain capillary perfusion and then the effectiveness of RCP for cerebral protection [54,55,57].

However, at the present time, no significant differences are reported between ACP and RCP in terms of mortality and permanent neurological complications [56], whereas it has been shown that ACP had a lower risk of transient cerebral adverse events [58].

## 7. Monitoring Techniques Associated with Possible Therapeutic Strategies

The monitoring and visualization of end organ oxygen supply and blood flow are of the utmost importance, and bilateral monitoring can assure clinicians that a sufficient supply of oxygenated blood is reaching both hemispheres of the brain.

Several studies suggested that a longer duration of deep hypothermic circulatory arrest is associated with neurocognitive impairment, but perioperative seizures, motor deficits and brain damage evident on magnetic resonance imaging (MRI) are consistent with anterograde cerebral perfusion (ACP) as well [8,59].

The question of an effective distribution and ideal quantity of cerebral blood flow, particularly in the contralateral left hemisphere, is one of the main issues about unilateral ACP.

As a matter of fact, effective neuromonitoring prevents negative consequences of a suboptimal cerebral perfusion [60,61].

Cerebral oximetry monitoring using near-infrared spectroscopy (NIRS) estimates regional hemoglobin oxygen saturation of blood in the brain (rSO_2_), taking advantage of the different absorptive properties of saturated and unsaturated hemoglobin (Hb) in the near-infrared spectrum. This method for cerebral oxygenation monitoring employs disposable sensors with an integrated near-infrared light source and photodetector placed on each side of the patient’s forehead. Sensor positioning allows one to monitor the ischemia-susceptible cortical tissue served from the anterior and middle cerebral arteries. However, its reliability in the monitorization of posterior territories’ oxygen imbalance or perfusion abnormality is low [62]. Moreover, NIRS may detect cerebral perfusion only for 3–4 cm of depth but gives little information about perfusion of the deeper brain regions.

Currently, rSO_2_ is a non-invasive method to continuously monitor changes in the local brain oxygen balance. Many reports have revealed that the monitoring of rSO_2_ allows early recognition of hypoperfusion and the subsequent performance of interventions to prevent prolonged rSO_2_ desaturation with the aim of avoiding neurological complications [62,63].

There are no defined normal or abnormal values for regional cerebral oxygen saturation. The most widely adopted definition for brain desaturation is a reduction of the rSO_2_ area under the curve (AUC) >20% of baseline value, or rSO_2_ under 50% of absolute value [61,64,65].

Supplemental cerebral perfusion is routine during aortic arch surgery: because of the hemodynamic characteristics of acute type I aortic dissection, cerebral perfusion is usually supported through a right axillary/innominate perfusion cannula. However, cerebral perfusion might not be symmetrically supported with unilateral selective anterograde cerebral perfusion (SACP), and hemispheric rSO_2_ differences might herald an inadequate left hemisphere perfusion [51]. This phenomenon can be caused by [66]:

(a) A displaced cannula.
(b) An arterial line obstruction.
(c) An interrupted circle of Willis.

Indeed, sudden decreases in rSO_2_ should trigger investigation of a possible mechanical obstruction (cannula dislodgment or arterial line compromise) [67]. If an interrupted circle of Willis is suspected when using unilateral SACP due to the left hemispheric reduction of rSCO_2_, bilateral ACP should be considered [11]. 

NIRS can be useful also to detect the opposite situation, as hyperperfusion and cerebral hyperoxia may be deleterious as well [68,69,70,71,72]: persistent hyperemia induces a vasogenic edema that may produce vasogenic edema, the major determinant of the cerebral hyperperfusion syndrome characterized by migraine symptoms, delirium, focal neurological deficit, seizures and coma [70]. 

The syndrome may develop with “normal” blood pressure and may be undetectable by tomographic brain imaging [71]. In this case, cerebral oximetry has been reported to be very helpful by Ogasawara et al. (2003) in the detection of hyperperfusion. They found the incidence of SPECT-confirmed pathologic post-endarterectomy hyperperfusion to be 12%: these authors showed cerebral oximetry to have 100% sensitivity and specificity in detecting this hyperperfusion [72]. Other investigators have reported on the value of rSO_2_ in detecting hyperperfusion accompanying antegrade cerebral perfusion during aortic arch surgery.

In order to induce cerebral vasodilation for homogenous cerebral tissue cooling, a modified alpha-stat strategy with carbon dioxide partial pressure (pCO_2_) elevation around 50–60 mmHg is routinely used [73,74]. We believe that the initiation of “full-flow” bypass over the innominate artery might be responsible for early hyperperfusion especially of the right hemisphere, which may explain why an alpha-stat strategy with a limitation of cerebral vasodilation is beneficial in these patients to avoid excessive overflow.

Initial “overperfusion” of the right hemisphere seems to persist during cooling despite the introduction of distal aortic perfusion (if performed with the same pump head) and the adjustment of blood pressure between both arterial lines [75].

In the current era, NIRS has become the pulse oximetry of perfusionists, cardiac surgeons and cardiac anesthesiologists; nevertheless, some drawbacks and barriers should be acknowledged in the context of aortic surgery, especially for type A dissection:

(a) Preoperative central venous saturation (ScvO_2_) concentrations are reflective of baseline severity of cardiopulmonary dysfunction, associated with short- and long-term mortality and morbidity, and may add to preoperative risk stratification in patients undergoing cardiac surgery. However, patients with aortic dissection have different clinical presentations which might significantly affect their baseline ScvO_2_; therefore, the role of the preoperative status on the risk of postoperative neurological dysfunction, and the most appropriate CPB and ACP strategies to match these findings are still open questions.
(b) rSO_2_ determined by NIRS is directly related to cerebral blood flow and jugular venous oxygen saturation (ScvO_2_); the innominate vein is sometimes interrupted during aortic surgery and this phenomenon might play a role in brain perfusion, especially in the left hemisphere during unilateral RCP [12,51].

Despite the strong pathophysiological and clinical rationale, NIRS has not been associated with an improvement in neurological prognosis in multiple studies [62,67].

Several explanations can be advocated [12,76]:

(a) NIRS is diagnosis, not therapy.
(b) There is no consensus about normal and abnormal values; indeed, validated thresholds are urgently required in aortic surgery (less than 50% or reduction more than 20% from baseline).
(c) There is no consensus about effective interventions.
(d) NIRS does not measure all the cerebral brain oxygen saturation, but only a small portion of the frontal cortex (average depth 2.5 cm): cortical atrophy gets the cortex away from the skull (less reliable in high risk elderly patients), and postoperative neurologic damages are not exclusively related to the frontal cortex oxygen supply reduction/imbalance.
(e) NIRS value can be influenced by non-brain sources (melanin, water).
(f) Ice packs are placed around the head of patients until the start of rewarming, which might jeopardize the reliability and reproducibility of the data.

In conclusion, the failure to make an explicit distinction between outcome prediction and outcome modification is key for the appropriate use of NIRS technology in aortic surgery [77,78]. If there is no “window of opportunity” to modify the outcome, despite being capable of predicting an adverse neurological outcome with a high degree of specificity and sensitivity, it is arguably of little clinical use; however, in aortic arch surgery, it should trigger interventions to optimize brain perfusion both mechanically (cannula repositioning, bilateral cannulation) and physiologically (temperature, CO_2_ management, drugs). In this regard, the technology should be part of the routine armamentarium of the aortic team [53,79].

Furthermore, NIRS monitoring and cerebral oxygen saturation levels during rewarming are associated with a delayed awakening time: this new indicator of postoperative delayed awakening after total aortic arch replacement reinforces that monitoring cerebral oxygenation early and throughout the rewarming phase may help to avoid a new clinical entity associated with ACP.

## 8. Cerebral Perfusion Monitoring: Integrative Strategies and Future Perspectives

### 8.1. Transcranial Doppler

The Transcranial Color Doppler (TCCD) is a useful tool for blood flow evaluation in large intracranial vessels (i.e., middle cerebral artery). This technique is sensitive for micro- and macro-emboli detection during aortic arch surgery [80]. The Transcranial Doppler allows for the measurement of cerebral blood flow and its distribution [81], giving the opportunity to optimize anterograde cerebral perfusion during circulatory arrest. In particular, for patients with an incomplete circle of Willis, when an inadequate cross-filling is detected, a shift from unilateral to bilateral cerebral perfusion could be promptly performed [82]. Moreover, the TCCD, differently from NIRS, allows a real-time detection of cerebral blood flow, possibly optimizing the perfusion strategy earlier [83]. The accuracy of the Transcranial Doppler depends on the quality of the images acquired through the transtemporal window (often challenging), to a stable position during monitoring and the sonographer expertise. The validity of Transcranial Doppler monitoring during selective anterograde cerebral perfusion needs confirmation in larger studies, and it is to date considered an integrative monitoring system used with NIRS.

### 8.2. Frequency-Domain Near-Infrared Spectroscopy (FDNIRS) and Diffuse Correlation Spectroscopy (DCS)

Frequency domain near-infrared spectroscopy and diffuse correlation spectroscopy represent the latest evolution in terms of optical techniques. These methods are more advanced than near-infrared spectroscopy for monitoring regional cerebral oxygen saturation. These techniques are based on estimates of the optical properties of tissues in relation to Hb concentration [84] and blood flow [83], respectively. In addition to the measurement of cerebral oxygen saturation, they also provide quantitative measures of cerebral blood volume and cerebral blood perfusion, enabling the calculation of a quantitative index of cerebral oxygen consumption (iCBF) [85]. The use of DCS has been demonstrated to effectively measure iCBF in a neonatal cardiac surgery study [86]. The real-time measurement of iCBF potentially avoids cerebral hypo- and hyperperfusion during circulatory arrest in aortic arch surgery. The role of these techniques is still to be defined in cardiac anesthesia.

## 9. Conclusions

The pathophysiology of brain damage during aortic surgery is nowadays well-elucidated, with the identification of several critical points associated with the onset of the insult. The different brain protection strategies determined a significant improvement in the patients’ prognoses, allowing complex surgical operations with an acceptable mortality and morbidity rate. The development of multiple options for intraoperative brain function monitoring enabled the clinicians to expand the traditional, limited evaluation of the ongoing brain insult. In this regard, a meaningful gap still exists between the diagnostic possibility and therapeutic action.

Further studies are needed to identify the best technique for cerebral protection and to translate the intraoperative detection of the insult to its mitigation or treatment.

## Figures and Tables

**Table 2:** A summary of studies comparing anterograde perfusion techniques for elective and emergent aortic surgery.

Author	Year	Etiology			Allocation		Cannulation Site	Flow	Temperature		Perfusate Temperature
		ATAAD (n.)	Aneurysm (n.)	Total (n.)	DHCA	MHCA + SACP			DHCA	MHCA	
Kazui [38]	1989	16	5	21	10	11	Innominate artery + left CCA	600 mL/min	15 °C	25 °C	-
Tan [39]	2003	32	-	32	19	13	Innominate + LCCA	10 mL/kg/min	15.1 ± 3.1 °C	25 °C	25 °C
Di Eusanio [40]	2003	122	167	289	128	161	Innominate + LCCA	10 mL/kg/min	16.1 ± 2.8 °C	23.2 ± 2.6 °C	-
Mulller [41]	2004	28	14	42	12	30	RSA	400–700 mL/min	20 ± 2 °C	22 ± 2 °C	-
Harrington [42]	2004	1	41	42	22	20	Innominate + LCCA	8–12 mL/kg/min	15 °C	25 °C	15 °C
Sundt [43]	2008	48	246	294	220	74	RAA	10–15 mL/kg/min	16–18 °C (NP); 23 °C (Bldr)	25 °C	13 °C
Halkos [44]	2009	105	166	271	66	205	RAA	10 mL/kg/min	18 °C	23.2 ± 4.2 °C	18 °C
Wiedemann [45]	2012	207	-	207	116	91	uACP: R. subclavian/innominate bACP: subclavian/innominate + L. carotid	10 mL/kg/min	18 °C	25 °C	25 °C
Misfeld [46]	2012	220	339	585	220	365	uACP RCCA bACP uACP: Innominate + LCCA	10–15 mL/kg/min	22 ± 2 °C	UACP 24 ± 3 °C (NP) BACP 25 ± 4 °C	24 °C

ATAAD, acute type A aortic dissection; n., number; CCA, common carotid artery; LCCA, left common carotid artery; RSA, right subclavian artery; RAA, right axillary artery; uACP, unilateral anterograde cerebral perfusion; bACP, bilateral anterograde cerebral perfusion; DHCA, deep hypothermic circulatory arrest; MHCA, mid-hypothermic circulatory arrest.

**Table 3:** Studies comparing outcomes associated with deep hypothermic circulatory arrest (DHCA) versus mid-hypothermic circulatory arrest (MHCA) associated with selective cerebral perfusion (SCP).

Author	Year	Postoperative Stroke DHCA	Postoperative Stroke MHCA + SCP	Postoperative Transient Neurological Dysfunction DHCA	Postoperative Transient Neurological Dysfunction MHCA + SCP	Postoperative Mortality DHCA	Postoperative Mortality MHCA + SCP
Kazui [38]	1989	1%	0%	-	-	14.3%	9%
Tan [39]	2003	28.6%	9.1%	-	-	-	-
Di Eusanio [40]	2003	12.5%	6%	6.2%	8%	13.2%	9.9%
Mulller [41]	2004	0%	10%	-	-	17%	23%
Harrington [42]	2004	9.1%	0%	4.5%	2.5%	0%	15%
Sundt [43]	2008	9.1%	5.4%	-	-	7.2%	8.1%
Halkos [44]	2009	4.5%	3.9%	9%	4.3%	22.7%	8.7%
Wiedemann [45]	2012	23.2%	12%	0.9%	2.1%	25.8%	13.1%
Misfeld [46]	2013	14.1%	9%	12.7%	15.9%	11.3%	11.7%

DHCA, deep hypothermic circulatory arrest; MHCA, mid-hypothermic circulatory arrest; SCP, selective cerebral perfusion.

**Table 4:** Comparing unilateral versus bilateral anterograde cerebral perfusion.

Author	Year	ATAAD	Chronic	Number of Patients	ACP	Flow	Temperature
Zierer [47]	2012	655	347	1002	u-ACP 673, b-ACP 329	1.6 ± 0.2 L/min; 1.7 L/min	26–34 °C
Preventza [48]	2015	157	0	153	u-ACP 90 (58.8%), b-ACP 63 (41.2%)	10–15 mL/kg/min	22–24 °C
Urbanski [49]	2020	0	1000	1000	u-ACP	1.4 ± 0.3 L/min	31 °C
Angleitner [50]	2020	184	0	184	b-ACP: n = 91, 49.5%; u-ACP: n = 93	10–15 mL/kg/min	20–28 °C
Norton [51]	2020	307	0	307	using uni-ACP (n = 140) and bi-ACP (n = 167).	10 mL/kg/min	24–28 °C

ATAAD, acute type A aortic dissection.

**Table 5:** Studies comparing unilateral versus bilateral anterograde cerebral perfusion.

Author	Year	Stroke	Transient Neuro Dysfunction	Mortality
Preventza [48]	2015	13 of 88 u-ACP patients (14.8%) and 8 of 62 b-ACP patients (12.9%) had a postoperative stroke (*p* = 0.75)	10 pts u-ACP (11.4%) and 5 pts b-ACP (8.2%) patients (*p* = 0.53)	The operative mortality was 13.3% (n = 12) with u-ACP and 12.7% (n = 8) with b-ACP (*p* = 0.91)
Urbanski [49]	2020	u-ACP 1%	u-ACP 4.9%	30 days 1.3%, in-hospital 2.1%
Angleitner [50]	2020	u-ACP:19.4% (18 pts), b-ACP 18.7% (17 pts)	u-ACP 9.7% (9 pts), b-ACP 7.7% (7 pts)	30-day mortality u-ACP: 16.1%, b-ACP: 12.1%
Norton [51]	2020	u-ACP 2.8% (4 pts) vs. b-ACP 6.5% (11 pts)	u-ACP 3.5% (5 pts) vs. b-ACP 2.4% (4 pts)	30-day mortality: uni-ACP 3.4% vs. bi-ACP 7.8%, *p* = 0.12)
Zierer [47]	2012	u-ACP 4% (14/673) vs. b-ACP 2% (14/329)	u-ACP 4% (30/673 pts), b-ACP 4% (12/329)	30-day mortality u-ACP: 5% (32/673 pts), b-ACP 5% (15/329 PTS)

uACP, unilateral anterograde cerebral perfusion; bACP, bilateral anterograde cerebral perfusion; pts, patients.

## Data Availability

No raw data are analyzed in this review. We report only published data.
